# Neighbors help neighbors control urban mosquitoes

**DOI:** 10.1038/s41598-018-34161-9

**Published:** 2018-10-25

**Authors:** Brian J. Johnson, David Brosch, Arlene Christiansen, Ed Wells, Martha Wells, Andre F. Bhandoola, Amy Milne, Sharon Garrison, Dina M. Fonseca

**Affiliations:** 1 Center for Vector Biology, Rutgers University, New Brunswick, NJ USA; 2 Community Mosquito Control, Town of University Park, University Park, MD USA; 3 Mosquito Repression Program, Town of University Park, University Park, MD USA; 4 Walter Johnson High School student volunteer, Bethesda, MD, USA

## Abstract

The worldwide spread of invasive *Aedes* mosquitoes and arboviral disease, have renewed the pressure for effective and sustainable urban mosquito control. We report on the success of a model we are confident will usher in a new era of urban mosquito control. The key innovation is the mobilization of neighbors guided by scientific advisors, an approach we termed Citizen Action through Science (Citizen AcTS). This approach was tested in a NE US town of approximately 1,000 residential yards infested with the invasive Asian tiger mosquito, *Aedes albopictus*, a major nuisance arboviral vector. We report a highly significant reduction in biting pressure that was maintained over time, and establish the thresholds needed for success. The Citizen AcTS model rejects the top-down approach consistently associated with intervention failures. Instead, it works through respectful exchanges among scientists and residents that lead to trust and individual ‘buy-in’ and transferring program ownership to the community.

## Introduction

It has long been recognized that successful urban-*Aedes* control programs require community support and direct participation. This has been highlighted by the failures of vertically structured (top-down), government-led programs to control epidemic dengue^1^ and the spread of chikungunya and Zika viruses^2^. While worldwide, government and non-government organizations are increasing their use of citizen volunteers for large scale surveillance and other high-labor activities^3,4^, the use of active citizen participants in mosquito control is currently largely nonexistent likely because they have seldom been successful^5,6^. However, urban mosquitoes whose immatures develop almost exclusively in small containers in residential yards difficult to access and treat by state or county professionals^7^, are an especially logical target for citizen efforts. Failure of traditional citizen-based interventions centers on high participant attrition rates and lack of direct personal motivation. Poor long-term participation likely relates to the difficulty in addressing the diverse intrinsic and extrinsic motivating factors driving initial and sustained volunteer participation^8^. Initial participation depends largely on personal interest and awareness of social responsibility, whereas long-term participation often relies on cultivation of strong relationships between the volunteers and scientists, and between volunteers and their communities^9^. The Citizen Action Through Science (AcTS) model was developed in an effort to exploit these motivating factors to improve initial adoption and long-term sustainability. Citizen AcTS employs a ‘buy-in’ model wherein individual residents purchase, deploy and maintain lethal oviposition traps (i.e., traps targeting and killing egg-laying females), and transfers program ownership to the volunteers and community leaders who are mentored by, and work closely with, scientific advisors.

Initial development of the Citizen AcTS model began during discussions between Rutgers scientists and residents of the city of University Park, MD (USA) who were afflicted by *Aedes albopictus*, the Asian tiger mosquito, a high nuisance and also important vector species^10,11^. Community volunteers, which subsequently created the University Park Community Mosquito Control, were advised to consider a mass-trapping intervention targeting females ready to lay eggs (gravid), which are responsible for propagating the next generation of biting adults. This recommendation was supported by a series of mass-trapping interventions by the Center for Disease Control and Prevention (CDC) in Puerto Rico that resulted in long-term reduction (over 80%) in *Aedes aegypti* densities^12,13^. These reductions were subsequently linked to a 50% decrease in human chikungunya exposure during the 2014 outbreak^14^. Of note, while all mass-trapping interventions of this nature had targeted *Ae. aegypti* in resource-poor or tropical settings^15^, we proposed that a community based mass-trapping intervention would be effective in controlling temperate *Ae. albopictus* because (a) the simplicity and direct association to an understandable mosquito behavior (oviposition) and control outcome (i.e., observable dead mosquitoes) make trap-based interventions an excellent “teaching tool” that maintains participant enthusiasm; (b) the relatively inexpensive but real investment in the traps (approximately 15–20 USD each) instills personal interest in the outcome, which is maximized by cleaning and otherwise maintaining the traps and removing competing containers; and (c) in temperate climates mass deployments can be focused in a relatively short period of time in late spring to prevent exponential population growth. We advised residents to purchase Gravid *Aedes* Traps (GAT), a large, passive oviposition trap successful in capturing urban *Aedes* mosquitoes^16^. This was confirmed with a pilot trial in the study area in 2016 (Table 1) during which *Ae. albopictus* accounted for >90% of mosquitoes collected. Through an arrangement with the manufacturer, we made GATs available at cost through Rutgers University in exchange for address, phone and email contact information allowing us to create an accurate map of the location of each lethal oviposition trap. Further, due to the potential for insecticide resistance^17,18^ and resident concerns regarding insecticide exposure, we advised that canola oil be used as the killing agent^19^. The use of canola oil, a non-toxic agent and common food staple, increased the acceptance of this approach by the residents. Of note, sticky papers inside the transparent dome, which were not available in 2016, now provide an alternative killing method also non-toxic that are easier to use.

**Table 1:** Summary of urban mosquitoes collected in GATs in University Park, MD during August of 2016.

	*Aedes albopictus*	*Aedes triseriatus*	*Aedes japonicus*	*Culex*/other
Total Collected (no. traps = 174)	1049	99	7	10
Trap Average (SD); 1 week trap interval	5.9 (7.5)	0.56 (1.8)	0.04 (0.22)	0.06 (0.25)
Percentage of Collection	90.04	8.49	0.60	0.86

**Figure 1:**
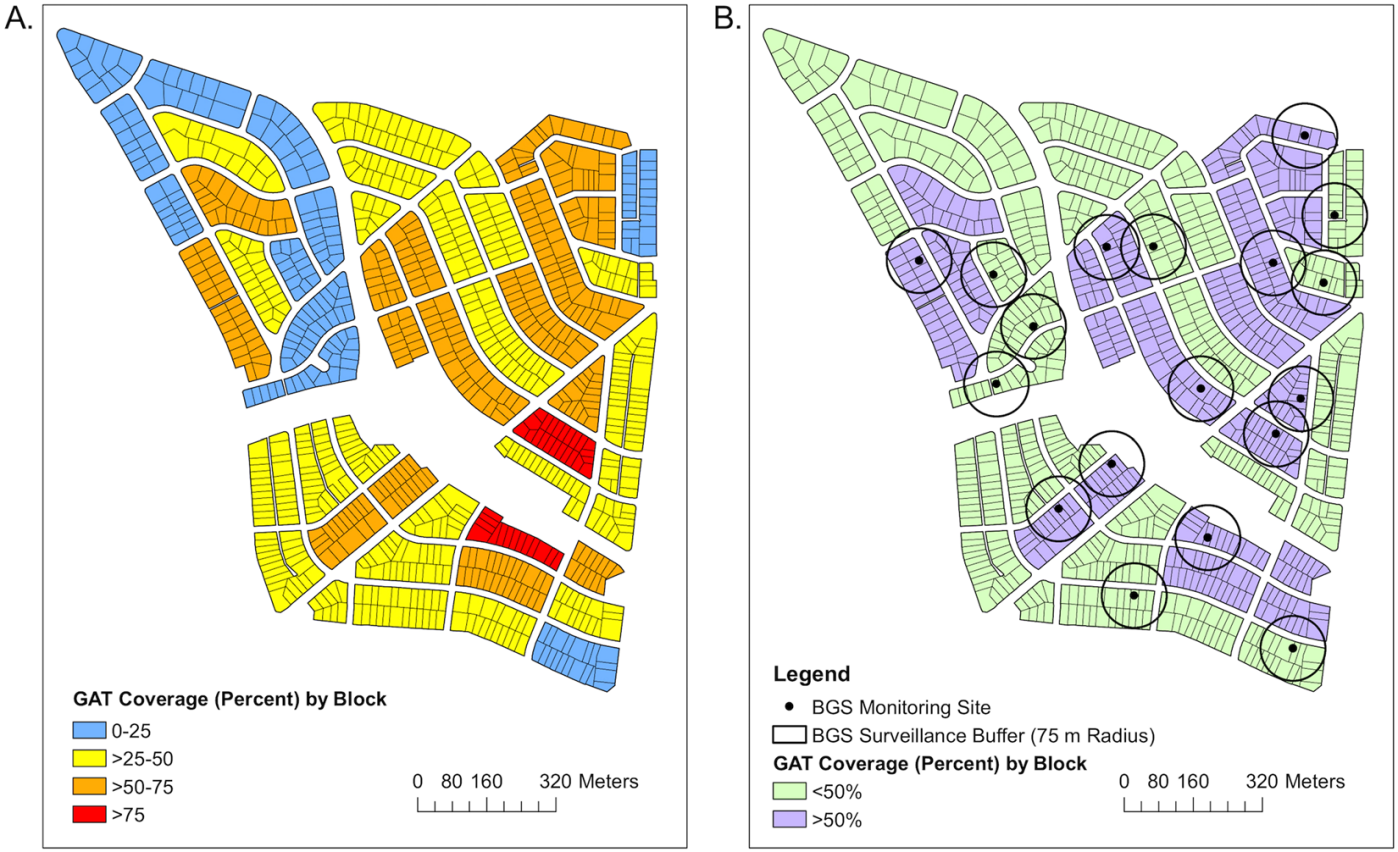
(**A**) Individual Gravid Aedes Trap (GAT) coverages (percentage of yards with traps) for all blocks in University Park, MD and (**B**) selected high (>50%) and low (<50%) coverage adult *Ae. albopictus* surveillance blocks. Participating households purchased 2 GAT traps; one for the front yard and one for the backyard. BGS refers to the BioGents Sentinel trap used to monitor host-seeking female abundance for research results^29^. To protect the privacy of the residents the specific locations of the yards with GATs are not visualized but de-identified data are available upon request.

After the initial pilot study developed during the summer of 2016, a city-wide campaign was initiated in early 2017. Each interested resident was encouraged to acquire 2 GATs, one for the front yard and one for the backyard: http://vectorbio.rutgers.edu/CitizenAcTSMD.htm. Traps obtained through the website were distributed with written instructions during several community wide events where live demonstrations on how to assemble and treat the trap with canola oil and make the infusion water (attractant), as well as the use of the biologic *Bti*^20^ to kill any developing larvae in the water, were also performed. Supplemental educational materials and project information were available through a dedicated website developed by the community (https://sites.google.com/site/mosquitocontrolup/mosquito-collection), through mass email campaigns and “word of mouth”. Individual city blocks were canvassed by community leaders referred to as “block captains” that educated their neighbors about the initiative and trap operations. Trap distribution began in late June with >95% of traps being purchased prior to the summer peak in *Ae. albopictus* abundance^7^. A total of 46% of potential residential yards (439 out of 954) deployed GATs (Fig. 1). This total includes 411 unique participants (yards) in 2017 and 28 carryover participants from 2016. The majority of participants (89.3%) obtained the suggested two traps while smaller percentages obtained more than two (up to six, 7.4%) or a single (3.3%) trap. After purchase, participants were fully responsible for setting and maintaining the GATs. At the end of the study, a single block survey (29 homeowners) was performed by one of the block captains to assess his neighbor’s perception and level of participation during the intervention. The block captain designed and performed the survey voluntarily, which highlights the kind of intellectual and social capital that is freely available in engaged communities.

Of note, residents of University Park were overall well aware of the need to remove, empty or treat water containers in their yards: since 2012 the town of University Park has supported a source reduction education campaign titled “Take Back Our Yards” aimed at educating residents about mosquito behavior and the importance of source reduction as a means of urban mosquito control. In 2017 the town intern also provided information about lethal oviposition trap deployment. The removal of other containers with standing water maximizes the likelihood that *Aedes* females will attempt to lay eggs in the GAT, killing them and all their progeny. Importantly, if a GAT is present in an otherwise clean yard, female *Ae. albopictus* may also be less likely to lay eggs in cryptic habitats harder to identify and remove or treat^7^.

Perception of the intervention, assessed through a survey just among neighbors in a HIGH coverage block and therefore not representative of the average resident experience, was overall positive, with the majority of survey respondents (43%, Table 2) noting a reduction in mosquito populations relative to the previous year; however, 26% noted no difference and the rest had no reference (e.g., did not live in the area the previous year). Despite differing perceptions, all survey respondents plan to deploy the traps in 2018. Although monthly trap maintenance intervals were recommended, actual intervals varied with the majority of respondents either maintaining their traps ‘occasionally’ or as recommended, with smaller percentages maintaining traps at shorter intervals or not at all. This result suggests that additional engagement activities focused on the importance of trap maintenance may lead to further reductions in *Ae. albopictus* populations.

**Table 2:** Description and summary of block survey. A total of 29 residents within a single block were contacted and surveyed by their block captain. Of the 29 residents, 23 completed the full survey.

Summary of Response Pool		
Survey Response	Number of Reponses	Percent
Responded to survey in full	23	79.3%
Put out traps and had yard survey, but moved away during the summer	1	3.4%
Put out traps and had yard survey, but did not live in the house due to remodeling construction	1	3.4%
Declined to participate	1	3.4%
Unable to contact owners	3	10.3%
**Questions/Responses**	**Number of Participants**	**Percent**
**Question 1**. Did you notice a difference in the number of mosquitos this year vs last year (or, before and after you put out traps)?		
Yes, fewer mosquitoes	10	43%
No, no difference	6	26%
No basis for opinion	7	30%
TOTAL	23	100%
**Question 2**. Since you put your traps out, how many times have you changed the water and Mosquito Dunks^®^?		
Every two weeks	2	9%
Monthly	6	26%
Occasionally	10	43%
Never	5	22%
TOTAL	23	100%
**Question 3**. Do you expect to put your traps out next year?		
Yes	23	100%
No	0	0%
TOTAL	23	100%

**Figure 2:**
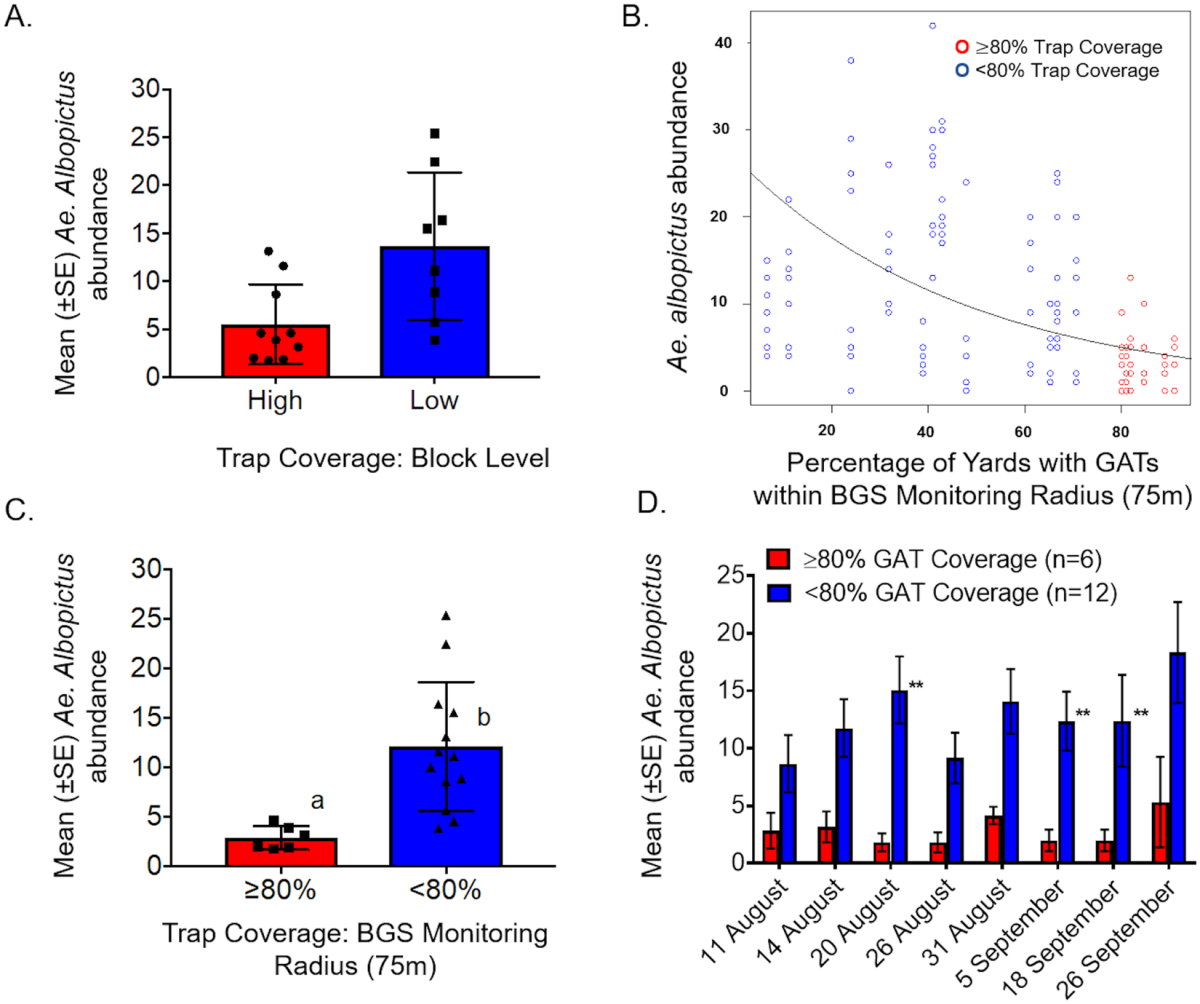
(**A**) Mean (±SE) female *Ae. albopictus* abundance at the block level (high >50% trap coverage; low <50% trap coverage) determined by the number of yards per block with GATs. (**B**) Negative binomial regression of the relationship between trap density and female *Ae. albopictus* abundance. (**C**) Mean (±SE) female *Ae. albopictus* abundance by high (≥80%) and low (<80%) trap coverage (percent of yards with traps) within a 75 m surveillance radius from BGS monitoring sites. (**D**) Mean (±SE) female *Ae. albopictus* abundance during each collection point in high (≥80%) and low (<80%) coverage monitoring sites. Different letters or presence of **indicate statistical significance between observations (*P* < 0.05).

The general results (Fig. 2A–D) reflect effective mosquito control solely accomplished through the cooperation of individual homeowners, community leaders, and scientific advisors without significant governmental organization or monetary support as the BGS trapping to assess effectiveness was developed with donations from the residents and a high school volunteer. Impact was consistent and directly relatable to degree of participation (i.e., reductions in biting pressure were greatest in areas of highest local GAT coverage) supporting the importance of achieving neighborhood engagement in a trap-based intervention^15^. The data also revealed a high degree of spatial and temporal variability in biting pressure across a wide-range of trap coverages. These differences are likely related to the fact that a few residents hired private mosquito control applicators to apply insecticides every 1 or 2 weeks over the entire summer (personal communications) and the existence of a long-running town-driven mosquito reduction information campaign. This variability also highlights the ability of urban *Aedes* to exploit small pockets of water, often ephemeral and cryptic^21^, that can undermine even effective source reduction attempts. It is important to note that a lack of accountability (i.e., improper trap setup and regular maintenance) will result in some degree of suboptimal participation regardless of resource level, as evidenced from the range of trap maintenance intervals observed in this study (Table 2). In order to maximize personal investment tangible outcomes such as seeing dead mosquitoes in the trap are needed to maximize participation while minimizing trap neglect. However, some degree of volunteer-based trap monitoring should be developed so that neglected traps do not become problematic, a task likely within the scope of identified ‘block captains’.

The success of the Citizen AcTs model relies on community-wide organization, neighbor canvassing and engagement, and individual investment, or ‘buy-in’, to encourage active rather than passive citizen involvement in projects that directly benefit them. This is in contrast to classical citizen science initiatives wherein participation is often spread out in space and time with individuals reporting an observation or other data point to a central organizing body or data repository^22^. Although standard citizen science has and will continue to provide great benefit to many scientific endeavors, relatively few programs have been successful in maintaining long-term volunteer participation^11^ and, most importantly, communication and cooperation among neighbors, a requirement of urban mosquito control. The Citizen AcTS model maximizes sustainability by relying on individual ‘buy-in’, tangible results, and transfer of program ownership to resident volunteers and community leaders. These factors appear critical to obtaining a ‘tipping point’ threshold of participation over which significant and persistent control is achieved. This threshold appears to center around 80% (Fig. 2B), an observation supported by similar but government organized initiatives in Puerto Rico^12,13^. Perhaps most importantly, the Citizen AcTS model does not rely on vertical structuring (top-down organization) so often associated with intervention failure^1,23^. In contrast, Citizen AcTS relies on community-driven bottom-up organizational structuring^24^. Although previous bottom-up interventions have generally failed to prevent epidemic dengue, there have been successes in mosquito control^25,26^ and, based on the results presented here, those willing to implement a mass-trapping intervention should consider adopting a similar model. Community-based approaches to mosquito control have the additional advantage of being the most cost-effective over the long-term^23^, particularly since economies of scale will drive the trap prices down as the market matures and expands. Moreover, by providing high-quality supporting information towards a shared focus, such as reducing biting mosquitoes and risk of mosquito borne diseases, Citizen AcTS increase sharing and trust among neighbors, essential for obtaining the level of collective action and social capital necessary to achieve self-sufficiency and long-term sustainability^27^.

## Methods

### Mosquito Population Monitoring

Adult host-seeking females were sampled using Biogents-Sentinel (BGS) traps (Biogents, Regensburg, Germany) operated for 24 h using 12 V rechargeable batteries. The traps were set without CO_2_ but with proprietary aromatic lures (BG-Lure). We performed a total of 8 sampling events (24 hr each) over a six-week period separated in time by an average of 6.12 days, limited by the need to avoid rain events.

### Spatial Analysis of BGS Distributions and Observations

We used Moran’s I and Average Nearest Neighbor analysis (ArcGIS 10.1) to measure the spatial autocorrelation of BGS trap locations and mean *Ae. albopictus* abundance per trap to determine if spatial correlation existed in the dataset. The distribution of BGS traps was found to be non-normally distributed (*Z* = 3.04, *P* < 0.01); however, mean female and male counts per BGS were found to be randomly distributed (*Z* = 1.73, *P* = 0.08; Z = 0.02, *P* = 0.98), indicating no spatial bias in trap observations. The observed mean nearest neighbor distance among traps was found to be 161.3 m, whereas the calculated expected mean distance was 118.2 m. To test for hotspots where there were consistently high (or low) female, male, and overall mosquito numbers, we used the Getis-Ord Gi* statistic (ArcGIS 10.0) with a Euclidean distance computed from mean BGS observations. No significant hotspots were identified for either group. Because *Ae. albopictus* is commonly associated with urban green-spaces within suburban landscapes^28^, we used linear regression to determine if female abundance was positively or negatively related to mean distance from identified green-spaces. The mean distance to all green-spaces was calculated for each BGS monitoring location using the proximity analysis feature in ArcGIS 10.0. No significant (*r*^2^ = 0.11, *P* = 17) association was observed.

### Relationship between GAT Coverage and Abundance of host-seeking Ae. albopictus

We used a general linear mixed model to access the effect of treatment we tested the null hypothesis that the number of female *Ae. albopictus* caught in BG Sentinel traps was the same within blocks with low (<50%) and high (>50%) GATs coverage. Collection order was included as a first-order autoregressive function to account for the repeated measures nature of the data. BGS trap ID was included as a random factor to account for individual trap bias. To test for the effect of GAT density, defined as the percentage of residential yards with GATs within 75 m of each BGS trap, we used a generalized linear mixed model with a negative binomial distribution with log link. A negative binomial distribution was chosen due to the over dispersion of the data. The covariance structure for the repeated estimation of mosquito density per trap per week was a first-order autoregressive function and BGS Trap ID was used as a random factor to account for individual trap bias using a covariance component identity matrix. Statistical differences between weekly collections in high (≥80%) and low (<80%) BGS monitoring sites was determined by a 2-way ANOVA performed on transformed (square root) data using the Sidák correction for multiple comparisons.
